# New Type of Sodium Alginate-*g*-acrylamide Polyelectrolyte Obtained by Electron Beam Irradiation: Characterization and Study of Flocculation Efficacy and Heavy Metal Removal Capacity

**DOI:** 10.3390/polym11020234

**Published:** 2019-02-01

**Authors:** Gabriela Craciun, Elena Manaila, Daniel Ighigeanu

**Affiliations:** National Institute for Laser, Plasma and Radiation Physics, Electron Accelerators Laboratory, #409 Atomistilor St., 077125 Magurele, Romania; gabriela.craciun@inflpr.ro (G.C.); daniel.ighigeanu@inflpr.ro (D.I.)

**Keywords:** electron beam irradiation, grafting, flocculants

## Abstract

The goals of the paper were first the obtainment and characterization of sodium alginate-*g*-acrylamide polyelectrolytes by electron beam irradiation in the range of 0.5 to 2 kGy, and second, the evaluation of flocculation efficacy and heavy metal removal capacity from aqueous solutions of known concentrations. Based on sodium alginate concentration, two types of grafted polymers were obtained. Physical, chemical, and structural investigations were performed. Flocculation studies under different stirring conditions on 0.5, 0.1 and 0.2% kaolin suspension were done. The removal capacity of Cu^2+^ and Cr^6+^ ions was also investigated. The acrylamide grafting ratio on sodium alginate backbone was found up to 2000% for samples containing 1% sodium alginate and up to 500% for samples containing 2% sodium alginate. Transmittances between 98 and 100% were obtained using, in the flocculation studies, polyelectrolytes containing 2% sodium alginate in concentrations of 0.5 and 1 ppm on kaolin suspension of 0.1 wt %. The polymer concentration was found critical for kaolin suspension of 0.05 and 0.1 wt %. Polymers containing 1% sodium alginate were efficient in Cr^6+^ ion removal, while those containing 2% in Cu^2+^ ion removal.

## 1. Introduction

Polyelectrolytes are water-soluble polymer carrying ionic charge along the polymer chain. They can be anionic or cationic and are available in a wide range of charge densities and molecular weights [1,2]. For at least forty years, their main applications in water treatment have been coagulation, flocculation, and dewatering of the sludge in treatment plants [3,4]. There are some advantages to their use in water and wastewater treatment, by comparing with the classic alum usage, such as: Dose requirements are notably lower, the formed flocs are much larger and stronger, the resulted sludge volumes are smaller, the phase separation between solid and water is considerably increased, the range of wastewaters that can be treated is wider, and last but not least, costs are up to 25–30% lower [3,5,6]. There are also disadvantages to their use, such as higher costs in particular situations or the sensitivity to incorrect dosage, but the most important is connected with the environment due to their synthetic constituents [3,6]. The influence of molecular structures on coagulation/flocculation, on the rates of both precipitation and sedimentation, on product water quality and on the solids content of the final sludge is still of great interest [3,7,8]. In water and wastewater treatment, the first and simplest step for solid particles removal is by gravity. Because particles with diameters on the order of 10 µm are not settled down by gravity alone in an economically reasonable amount of time, a second process called *coagulation*, which consists of the destabilization of colloidal suspensions by neutralizing the electric forces that keep the suspended particles separated, is needed [9,10]. However, the aggregates formed in this process are small, strongly dependent on pH and its variations, and their sedimentation velocities are relatively low. The third process, called *flocculation*, which consists of the addition of inorganic/organic polymers, is necessary to be applied, sometimes even in conjunction with classical coagulants (salts of multivalent metals like aluminum and iron) [9,10]. Inorganic flocculants, very effective but usually used in very large quantities, leave large amounts of sludge and are strongly affected by pH changes. The organic flocculants, typically polymeric in nature, are not so effective, but some of them can be used even in ppm concentrations [9]. Among polymeric flocculants, the synthetic polymers can be tailor-made by controlling the molecular weight, molecular weight distribution, chemical structure of polymers, and nature and ratio of functional groups on polymeric backbone. Natural polymers, mainly polysaccharides, are fairly shear-stable in contrast with synthetic polymers, and biodegradable and easily available from reproducible farm or forest resources. The biodegradability of natural polymers reduces their shelf life and needs to be suitably controlled. It is, thus, evident that all polymers, whether natural or synthetic, have one or another disadvantage. Although attempts have been taking place for some time, the preoccupations to combine the best properties of natural and synthetic polymers are still topical [11,12,13]. Grafting is a useful method for modifying some properties of natural and synthetic polymers. In the grafting process [14,15,16], an amount of synthetic monomer is attached onto the polysaccharide backbone, the formed product providing properties from the base polymers and a number of new and favorable properties as shear stability [17,18,19]. It was envisaged [11,13] that by grafting flexible polyacrylamide chains on polysaccharides such as guar gum, xanthan gum, carboxy-methyl cellulose, and starch, it is possible to develop efficient, shear-stable, and potentially biodegradable flocculants for treatment of industrial effluents and mineral processing. In these flocculants, the flexible chains of polyacrylamide are grafted onto the rigid backbone of polysaccharides.

Even if the problem of colloidal suspensions or organic maters is solved by the existing methods, the presence of heavy metal still represents a challenge in the field of water and waste water treatment. These represent an ecotoxicological hazard of prime interest and increasing significance, because of their accumulation in living organisms [20,21]. For example, chromium and copper are very toxic metals even at low concentrations introduced into natural water from a variety of industrial wastes [20,21,22]. The chromium concentration that can be found in industrial waste water ranges from 0.5 to 270,000 mg/L, while the tolerance limit for the discharge of Cr(VI) in surface water is 0.1 mg/L, and in potable water 0.05 mg/L [20,21]. The permissible limit of copper ions in industrial effluents reported by USEPA is 1.3 mg/L and by WHO is maximum 2 mg/L [22]. A wide range of physical and chemical processes are available for both chromium and cooper ions removal from industrial wastewater, such as: Electrochemical precipitation, ultrafiltration, ion exchange, electrodialysis, reverse osmosis, chemical precipitation and adsorption for chromium and adsorption, cementation, membrane filtration, and electrodialysis or photocatalysis for cooper removal [20,21,22]. The major drawbacks with these processes are the high cost, toxic sludge generation or incomplete metal removal [20,21]. Because heavy metals cannot be biodegraded by microorganisms within the natural environment, the research and development of technologies that can remove these pollutants from water sources is currently a worldwide priority [23]. Consequently, there is an urgent need for the development of new flocculants and adsorbents having high flocculation and adsorption capacity and selectivity to remove heavy metal contaminants from aqueous media, and for this, grafted polymers are good candidates [24]. 

In the present study, the renewable and biodegradable alginate (a nontoxic, very user- and consumer-friendly polysaccharide) has been chosen to be grafted with polyacrylamide [25,26]. The goal of the paper is to present experimental results of the obtainment and characterization of a new type of polyelectrolyte, based on sodium alginate and acrylamide-sodium alginate-*g*-acrylamide. The electron beam irradiation in the range of 0.5 and 2 kGy was used to obtain the polyelectrolyte. Characterizations were made by means of various physical and chemical methods in order to determine the conversion coefficient, residual monomer content, intrinsic viscosity, molecular weight, and radius of gyration. The radiation effect was evaluated through the Fourier transform infrared spectroscopy technique. By connecting the results with the behavior of water and acrylamide in radiation field in order to obtain polyacrylamide that is grafted on the alginate backbone, a possible reaction mechanism between monomer, polymer, and solvent was proposed. The polymer flocculation efficacy in terms of transmittance against distillated water was investigated by flocculation studies on kaolin suspension of 0.05, 0.1, and 0.2% under different stirring conditions. The use of the polyelectrolyte as a single metal system in order to remove heavy metal ions of Cu^2+^ and Cr^6+^ from water solutions having known concentrations was also a goal. The residual metal ion concentrations in the solutions after the flocculation tests were measured using the spectrophotometric method, and the results were expressed in terms of removal efficiency and absorption capacity after 24 h. 

## 2. Experimental Section

### 2.1. Materials

The materials used in experiments are shown in Table 1. Acrylamide (AMD), sodium alginate (ALg), and potassium persulfate (PP, used as initiator) were purchased from Sigma Aldrich (Redox Group Company, Bucharest, Romania) and were used without further purification. All other reagents used for polyelectrolytes preparation and characterization were of analytical grade and used as received. The Cu^2+^ and Cr^6+^ metal ions used in the metal ion removal studies were provided as CuSO_4_ and K_2_Cr_2_O_7_ by Sigma Aldrich also. These reagents of analytical grade were prepared with double-distilled water [27].

### 2.2. Experimental Installation and Sample Preparation

The polyelectrolytes were obtained in atmospheric conditions and at room temperature of 25 °C by electron beam irradiation using the ALID-7 linear accelerator of travelling-wave type that was built in the Electron Accelerators Laboratory from the National Institute for Lasers, Plasma and Radiation Physics, Bucharest, Romania. The optimum values of the electron beam (EB) parameters, namely peak current *I*_EB_ and EB energy *E*_EB_, to produce maximum output power *P*_EB_ for a fixed pulse duration τ_EB_ and repetition frequency *f*_EB_ are as follows: *E*_EB_ = 5.5 MeV, *I*_EB_ = 130 mA, *P*_EB_ = 670 W (*f*_EB_ = 250 Hz, *τ*_EB_ = 3.75 μs) [27]. 

The performance of irradiation process depends on the strict control of the absorbed dose (*D*) and absorbed dose rate (D*) [27,28]. In our experiments, the used electron beam dose rate was 3.5 kGy/min in order to accumulate doses between 0.5 and 2 kGy. For radiation dosimetry, the primary standard graphite calorimeter was used. The EB penetration depth was calculated in order to assure equal doses at the entry and exit of the irradiated samples. The thickness of a sample as a function of EB energy and sample density resulted as being 2 cm, using the following formula [27,29]:

(1) E=2.6×t×ρ+0.3

where *E* [MeV] is the EB energy (5.5 MeV), *t* [cm] is the sample thickness, and *ρ* [g·cm^−3^] is the sample density, in our case 1 g·cm^−3^.

Two types of monomeric solutions (AMD/ALg I, containing 1% sodium alginate, and AMD/ALg II, containing 2% sodium alginate) were prepared and disposed for irradiation in polyvinylchloride (PVC) containers of a 3 cm diameter containing 15 mL of monomeric solutions.

### 2.3. Sample Characterization

#### 2.3.1. Physicochemical Characteristics

To determine the conversion coefficient (CC) and the residual monomer concentration (M_r_), 2 grams from each polymer called AMD/ALg I/0.5 to 2 and AMD/Alg II/0.5 to 2 were placed in 200 mL distilled water for 24 hours, then stirred for 1 hour at 400 rpm for a very good mixing. CC and M_r_ were determined using the titrimetric method, in which bromine reacts with the double bond of residual monomer. After complete dissolution in water, the polymers were treated excessively with a bromide-bromate solution, and the bromine excess was determined by means of the iodatometry method in the presence of sodium thiosulfate solution (1 M) [30,31,32]. The intrinsic viscosity (η_intr._) was determined using the falling ball Hoppler viscometer of BH-2 type [30,33]. The measured parameter is the ball falling time in the cylindrical tube inclined with 10 degrees against the vertical plane and filled with the liquid to be analyzed. The ball falling time through the polymeric solution was measured in five different concentrations. The working temperature was 30 °C and sodium nitrate 1N (NaNO_3_) was used as solvent. 

The relative viscosity was calculated using the following relation:

(2) $$\eta_{\mathrm{rel}}=\frac{t}{t_{0}}$$

where *t* is the falling time of the ball through the polymeric solution and *t*_0_ is the falling time of the ball through the solvent.

The specific viscosity was calculated from the relation:

(3) $$\eta_{\mathrm{sp}}=\left(\eta_{\mathrm{rel}}-1\right)$$

The reduced viscosity was determined using the relation:

(4) $$\eta_{red}=\frac{\eta_{\mathrm{sp}}}{c}$$

where *c* is the polymer concentration (g/dL). 

From the graphical representation of the η_red_ as a function of the copolymer concentration, the intrinsic viscosity η_intr_ was obtained by extrapolation. 

#### 2.3.2. Purification of Grafted Polymers and Grafting Reaction Evaluation

Different concentrations of polymerized mixtures from each sample were completely diluted in water, then added dropwise into a large excess of methanol (150 mL) in order to remove the homopolymer. The precipitated polymer was filtered off and washed with methanol 10 times [30,34]. Afterwards, it was precipitated by adding 250 mL of acetone in order to separate the unreacted monomer (acrylamide) from the grafted polymer, and finally it was dried in a hot air oven at 60 °C for 6 h. The grafting ratio (%) and grafting efficiency (%) were calculated using the following relations [30,35,36,37]:

(5) $$Grafting\;ratio:\;GR\left(\%\right)=\frac{wt.\;of\;graft\;polymer-wt.\;of\;sodium\;alginate}{wt.\;of\;sodium\;alginate}\times 100$$

(6) $$Grafting\;efficiency:\;GE\left(\%\right)=\frac{wt.\;of\;graft\;polymer-wt.\;of\;sodium\;alginate}{wt.\;of\;monomer}\times 100$$

#### 2.3.3. FTIR Analysis and Reaction Mechanisms

The polyelectrolyte chemical structure was investigated using the TENSOR 27 FTIR Spectrophotometer (Bruker, Bremen, Germany) by ATR measurement method. All the spectra were the average of 30 scans realized in absorption in the range of 4000–600 cm^−1^, with a resolution of 4 cm^−1^. By connecting the grafting reaction evaluation with the spectral analysis results, a reaction mechanism was realized. 

#### 2.3.4. Flocculation Studies

Flocculation studies were carried out on kaolin suspension, at room temperature of 25 °C using the standard Jar test apparatus of Velp FC 6S type. The jar test apparatus having 6 stirrer blades that can rotate with a variable speed between 10 and 100 rpm in 6 beakers of 500 mL was used in order to investigate the influence of the kaolin concentration (0.05, 0.1, and 0.2 wt %), polymer concentration (0.5 to 10 ppm), and rotation speed (60 and 90 rpm) on the flocculation efficacy. Every sample was stirred for 15 min and then left to rest for another 15 min before sampling for analysis. Clear supernatant was drawn from the top layer (up to 1–2-cm depth), and its transmittance was measured at 620 nm using the Cary Bio-100 UV–VIS spectrophotometer (Agilrom Scientific SRL, Bucharest, Romania).

#### 2.3.5. Metal Ion Removal Studies

Metal ion removal experiments were performed at room temperature in aqueous solutions containing concentrations of 500 mg/L Cu^2+^ and Cr^6+^ ions. The metal ion concentrations in the solutions after treatment were measured by means of the spectrophotometric method using the Cary Bio-100 UV–VIS apparatus. The influence of the irradiation dose and sodium alginate concentration on removal efficiency and equilibrium absorption capacity was studied. The removal efficiency, *R*(%), and absorption capacity, *q_e_*, after 24 h were calculated using the following equations [38,39,40]:

(7) $$R\left(\%\right)=\frac{\left(C_{0}-C_{24}\right)\times 100}{C_{0}}$$

(8) $$q_{24}\left(mg/g\right)=\frac{\left(C_{0}-C_{24}\right)\times V}{W}$$

where *C*_0_ and *C*_24_ are the concentrations of metal ions in aqueous phase before and after 24 h of treatment (mg/L), V is the volume of the aqueous phase, and *W* is the amount of flocculant. 

## 3. Results and Discussions

Two types of aqueous solutions based on acrylamide and sodium alginate were prepared and subjected to electron beam (EB) irradiation in an atmospheric condition and at room temperature of 25 °C, in order to obtain grafted polymers having flocculation properties. Details concerning chemical composition and irradiation dose are given in Table 2. 

### 3.1. Physical and Chemical Characteristics of Sodium Alginate-g-acrylamide Polymers

**Figure 1:**
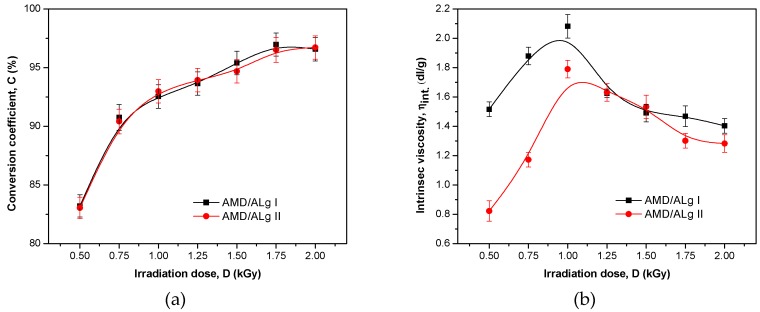
Conversion coefficient (**a**) and intrinsic viscosity (**b**) versus electron beam irradiation dose for AMD/ALg I and AMD/ALg II polymer types.

For the grafted polymers obtained as above, the conversion coefficient (CC), residual monomer concentration (*M*_r_), and intrinsic viscosity (η_intr._) were determined. The results are presented in Table 3 and Figure 1.

In Table 3 and Figure 1a, it can be seen that for both sodium alginate-*g*-acrylamide polymers, AMD/ALg I and AMD/ALg II, high conversion coefficients (up to 97% approximately) were obtained, correlated with low residual monomers contents (under 0.02%) as the radiation dose increased. The increase of the irradiation dose increased the probability of higher molecular contact, resulting in the propagation of active chain and continuous CC increase [14,30,41]. Notable differences between the samples having different initial concentrations of sodium alginate cannot be observed.

The intrinsic viscosity (Figure 1b) presented a difference of 88% between samples having 1% and 2% sodium alginate content, these results being favorable for the first category up to the irradiation dose of 1.25 kGy. At the irradiation dose of 1.25 and 1.5 kGy, both AMD/ALg I and AMD/ALg II polymer types presented almost similar values, not under the values obtained at 0.5 kGy. Between 1.5 and 2 kGy, the values split apart again but not as in the initial range.

**Figure 2:**
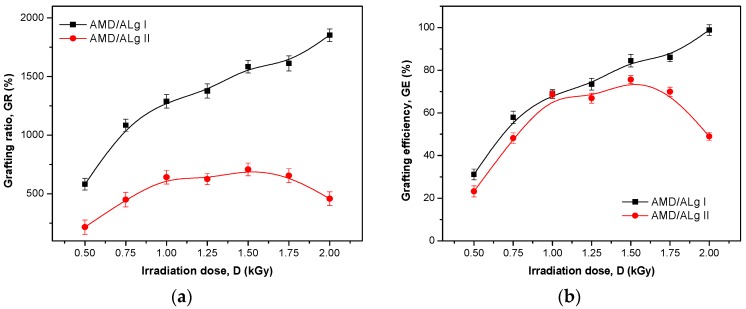
Grafting ratio (**a**) and grafting efficiency (**b**) as a function of sodium alginate concentration and irradiation dose.

The effect of sodium alginate concentration and irradiation dose on the grafting reaction was investigated and then correlated with the FTIR analysis. The grafting ratio (GR) and grafting efficiency (GE) were calculated and plotted and the results are presented in Figure 2.

As can be seen in Figure 2, doubling of the sodium alginate concentration leads to the decrease of both GR and GE. While the grafted polymers of AMD/ALg I type maintain an upward trend of both GR and GE, those of AMD/ALg II type presented maximum values at 1 and 1.5 kGy, and then GR and GE decreased. Thus, while the grafted polymers of AMD/ALg I type have GRs between 500% and 2000% depending on the irradiation dose, those of AMD/ALg II type just got up to 500%. The grafting efficiencies of both AMD/ALg I and AMD/ALg II types are comparable up to 1 kGy, being around 65–70%. Over this irradiation dose, the GEs of AMD/ALg I type increased up to 100%, while the GEs of AMD/ALg II type showed a plateau region between 1 and 1.5 kGy and then decreased.

As shown in Figure 1a and Figure 2, CC increased with the irradiation dose increasing for both AMD/ALg I and AMD/ALg II polymer types, while GR and GE did not continue to rise for AMD/ALg II. Thus, we can say that differences in GR and GE appeared because of doubling the sodium alginate concentration, as long as the concentrations of PP and AMD were the same. The difference between initial and final temperature after irradiation in samples was not over 20 °C, so we cannot say that the temperature effect stressed the strong influence of the nature of the radical with respect to their ability of monomer addition [41]. 

The intrinsic viscosity of a polymer, that is, the indicator of its hydrodynamic volume in solution, depends on the molecular weight, structure, chain dimension, and nature of the solvent, as well as the temperature of the medium. For two polymers having similar molecular weight, the branched polymer has a lower hydrodynamic volume compared to its linear counterpart and, thus, has lower intrinsic viscosity. Furthermore, long branches determine a higher intrinsic viscosity and vice versa [30,42,43,44]. In dilute solutions, the polymer chains are separate, so the polymer intrinsic viscosity depends only on the dimensions of the polymer chain and on the molecular weight [30,43,45]. Grafted polymers having high percentages of grafting efficiency will have the highest intrinsic viscosity because a higher percentage of grafting means longer polyacrylamide chains grafted onto the backbone of sodium alginate. With the increase of the absorbed dose, the intrinsic viscosity and grafting efficiency are enhanced, continuously achieving the maximum when the absorbed dose is between 1 and 1.4 kGy, and then both parameters decrease. This may be due to the accumulation of polyacrylamide molecules in close proximity to the sodium alginate backbone when the irradiation dose increases. The decrease of the intrinsic viscosity as well as of grafting efficiency after the optimization of the irradiation dose could be associated with the reduction of the active sites on the sodium alginate backbone [30,35]. 

### 3.2. Fourier Transform Infrared Spectroscopy (FTIR)

**Figure 3:**
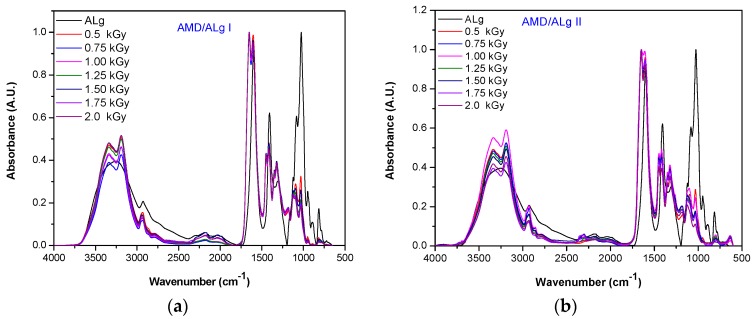

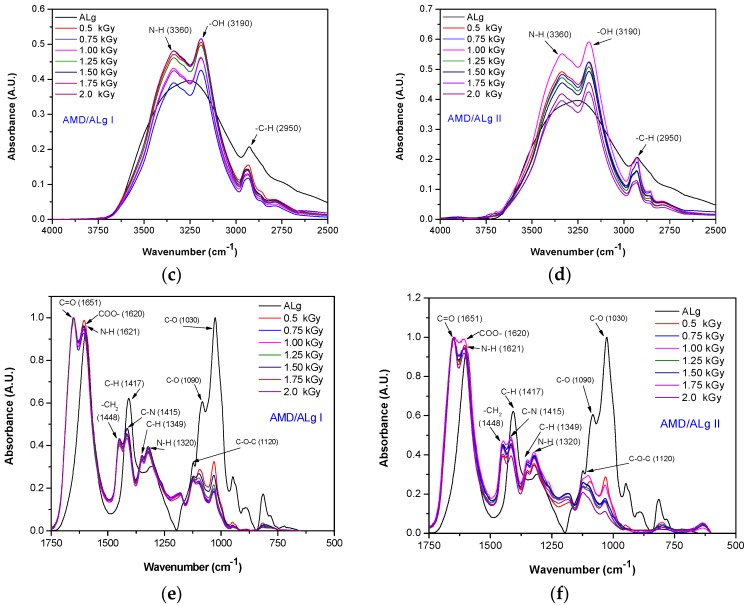
The FTIR spectra of AMD/ALg I (**a**) and AMD/ALg II (**b**) samples. Details of the AMD/ALg I (**c**), AMD/ALg II (**d**) spectra between 4000–2500 cm^−1^ and AMD/ALg I (**e**), AMD/ALg II (**f**) spectra between 1750–500 cm^−1^.

In order to evaluate the binding of acrylamide on the sodium alginate backbone, the infrared spectra of the grafted polymers were done. By comparing the results with those obtained by other tests, a reaction mechanism was realized and the best polymers obtaining conditions were established. In Figure 3, the entire (a,b) and detailed (c–f) overlapped spectra of the AMD/ALg I/ and AMD/ALg II/ samples are presented. 

The band near 3400 cm^−1^ that corresponds to the stretching vibration of –OH groups of sodium alginate [25,46,47,48,49] can be seen in both AMD/ALg I (Figure 3a,c) and AMD/ALg II (Figure 3b,d) spectra. On this band, modifications are observed in absorbance for samples obtained at the same irradiation dose as a function of sodium alginate concentration. For example, the samples obtained at the lowest irradiation dose present significant increases of the absorbance, while samples obtained at the highest irradiation dose present significant decreases of the absorbance (Figure 3c,d). The variation in intensity and the shifted appearance at 3192 cm^−1^ (Figure 3d) and 3191 cm^−1^ (Figure 3c), respectively, indicates the partial participation of hydroxyl groups in the chemical reaction [25]. By doubling the sodium alginate concentration in the irradiated samples, the intensities of all samples decreased, except for the intensity of AMD/ALg II/1 sample obtained by irradiation at 1 kGy (Figure 3d). This result indicates the formation of new bounds between –NH_2_ groups of polyacrylamide and carboxyl groups of alginate [49].

The sharp bands around 1620 cm^−1^ are attributed to the asymmetric COO-stretching. The bands at 1417 cm^−1^ (Figure 3e) and 1415 cm^−1^ (Figure 3f) and the bands around 1321 cm^−1^ (Figure 3e,f) correspond to the C–H deformation with secondary alcohols [49]. Finally, the bands around 1120 cm^−1^ (1122 cm^−1^ for both AMD/ALg I and AMD/ALg II), 1090 cm^−1^ (1094 cm^−1^ for AMD/ALg I and 1093 cm^−1^ for AMD/ALg II), and 1030 cm^−1^ (1032 cm^−1^ for both AMD/ALg I and AMD/ALg II) are due to the asymmetric C–O–C stretching, C–O stretching in the CH–OH structure and symmetric C–O stretching in the C–O–C structure, respectively (Figure 3e,f) [49]. 

The bands at 3360 and 1320 cm^−1^ are usually attributed to the stretching vibration of N–H [49] and we found them shifted at 3335 and 1321 cm^−1^, respectively. The same situation was observed in the case of the band corresponding to the C=O stretching (around 1651 cm^−1^ instead of 1670 cm^−1^). The bands corresponding to the N–H deformation for primary amine, CH_2_ in-plane scissoring, C–N stretching for primary amide, C–H deformation, and NH2 in-plane rocking were found at 1621, 1448, 1417–1415, 1349, and 1122 cm^−1^, respectively. 

By comparing Figure 3a,c,e with Figure 3b,d,f, it is easy to observe the differences in the intensities of the same bands. In this case, the sodium alginate addition in the polymer samples of AMD/Alg II type leads to the increase of the band intensity. These results are very well correlated with the results presented in Table 3 and Figure 1b and Figure 2a,b, in which the intrinsic viscosity, grafting ratio, and grafting efficiency are obviously affected by doubling the sodium alginate concentration in the samples of AMD/ALg II type.

**Figure 4:**
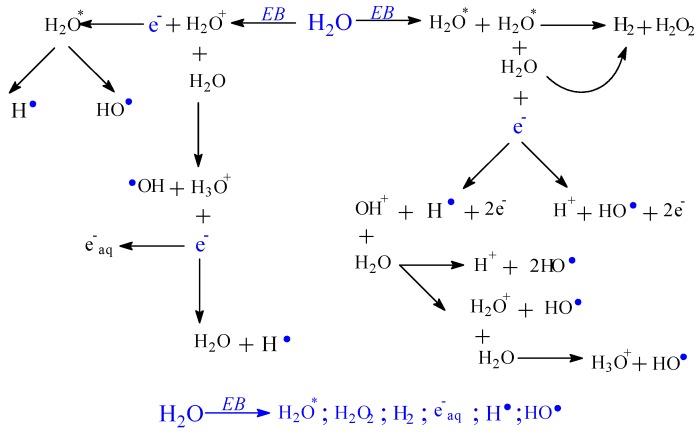
Possible mechanism for obtaining radicals by irradiation from water.

**Figure 5:**
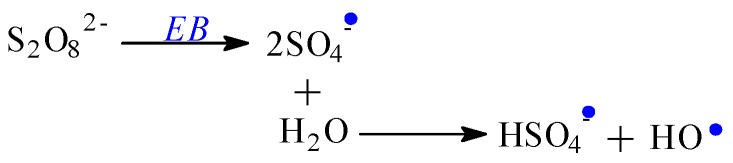
Possible mechanism for obtaining radicals by irradiation from initiator, potassium persulfate.

**Figure 6:**
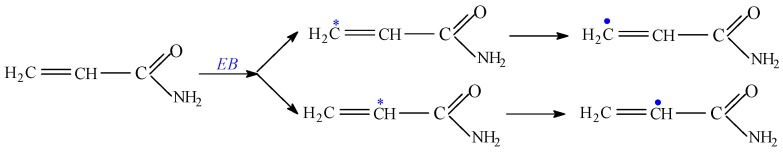
Possible mechanism for obtaining radicals by irradiation from acrylamide.

**Figure 7:**
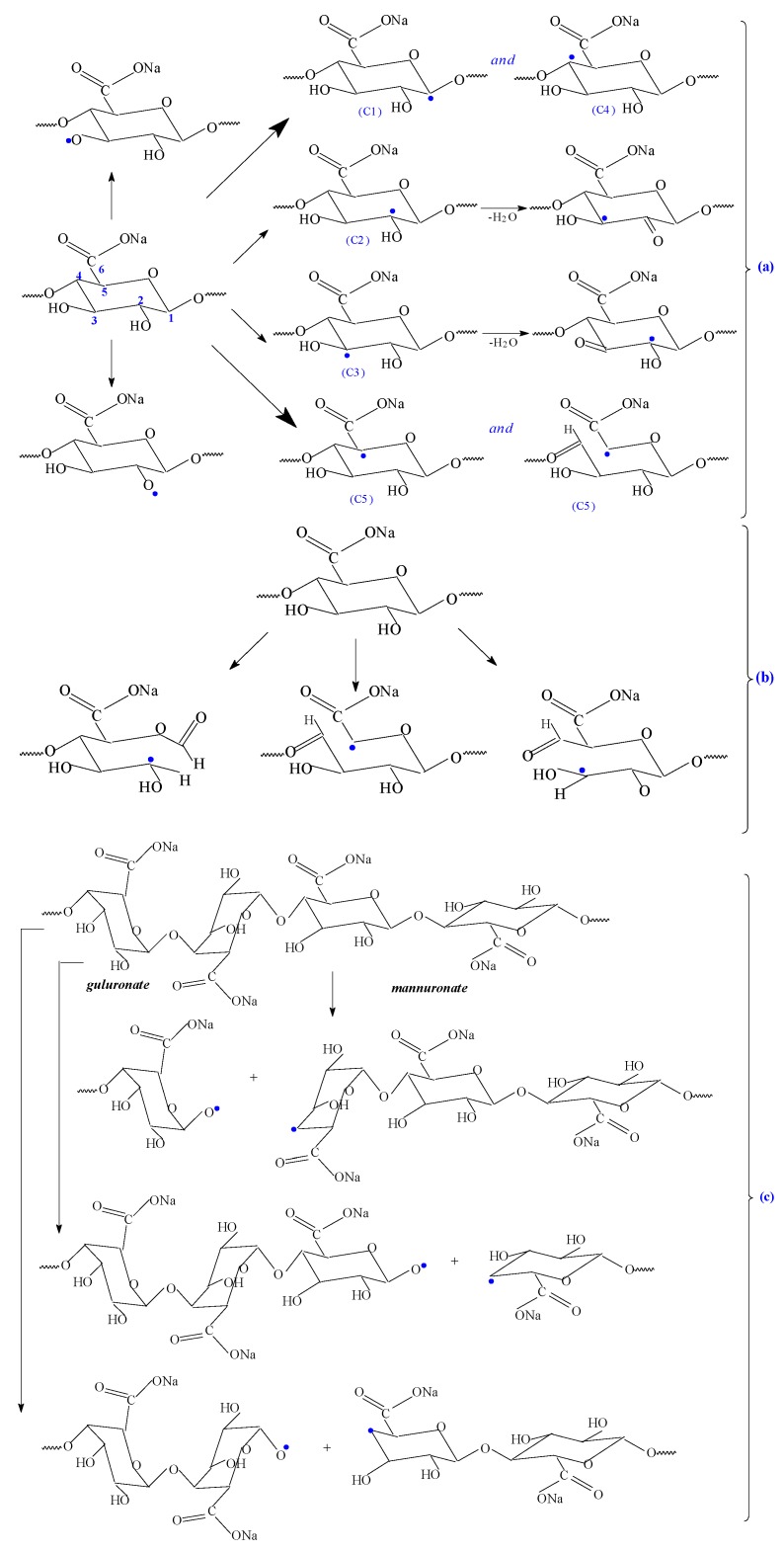
Possible mechanism for obtaining radicals by irradiation from sodium alginate.

The ionizing radiation effects on monomers and polymers are the production of polymerization, cross-linking, grafting, and degradation reactions. After the interaction with monomer or polymer molecules, the electron beam, as ionizing radiation, produces, in addition to ionization, the excitation of the active species [50,51]. The energies associated with these phenomena are strongly dependent on the radiation dose. Free radicals are formed by the dissociation of molecules in the excited state or by the interaction of molecular ions. These can react through a direct connection with the polymer chains but can also initiate grafting reactions [52,53]. The formation of radicals by the decomposition of all reactants (initiator, solvent-water here and monomers) constitutes an advantage in the radiation induced reactions. In Figure 4, Figure 5, Figure 6 and Figure 7, decomposition of the reactants from the system is presented: Solvent-water radiolysis (Figure 4), initiator–potassium persulfate (Figure 5), and monomers-acrylamide (Figure 6) and sodium alginate (Figure 7). Even if in radio-induced polymerization the initiation of the reaction is done by irradiation, the initiators as potassium persulfate are used in order to optimize the monomer (AMD) conversion process [54].

Chemical reactions presented in Figure 7a,b correspond to the mannuronate decomposition and are similar with the guluronate decomposition, the second group of sodium alginate.

The main role of the ionizing radiation is the achievement of the first step of the polymerization process, the initiation step, leading to the formation of free radicals. The next steps, propagation, completion, and the chain transfer, take place almost identically as in classic polymerization/grafting processes. Through the sodium alginate irradiation, radicals having unpaired electrons are formed, mainly in positions 1, 2, 3, 4, and 5 of the pyranose ring, as is shown in Figure 7. These radicals should originate from hydrogen abstraction from positions mentioned above and from the OH group [55]. The radicals formed from positions 2 and 3 of the pyranose ring may form, in their turn, other radicals by loss of a water molecule. The radical species can be also formed by cleavage of a glycosidic bond, from α and β-fragmentation of an oxygen-centered radical resulted from the cleavage of a glycosidic bond [55], and by chain scission, as shown in Figure 7c. As seen in Figure 7a–c, free radicals are formed after C–H, C–O, and C–C bond cleavages by hydrogen abstraction, chain scission, and cycle opening. Through acrylamide addition, no unsaturated bond remains when a radical is created.

**Figure 8:**
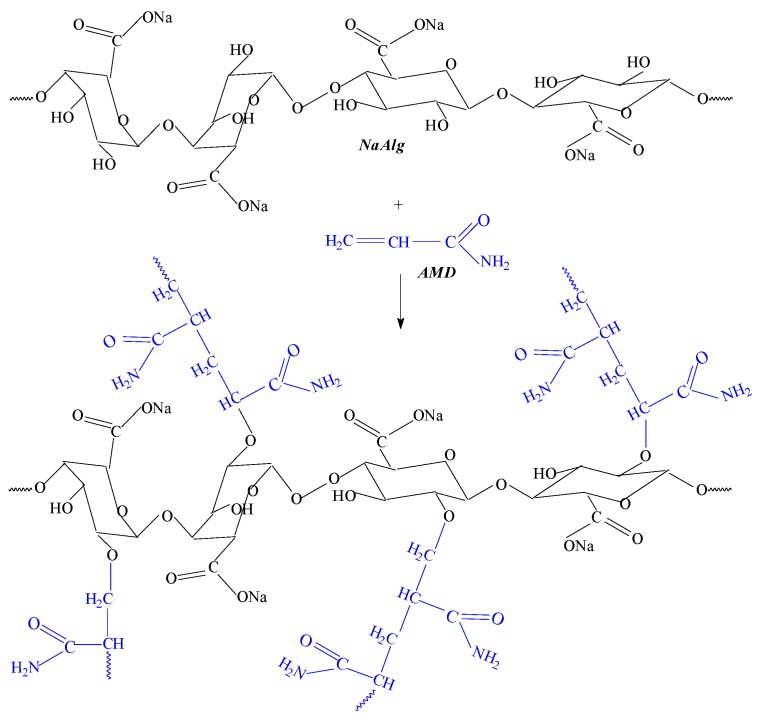
Possible mechanism of acrylamide grafting on sodium alginate backbone.

In order to correlate the results obtained by the FTIR technique with those obtained after the evaluation of the flocculation performance, based on other results presented in the literature [25,47,56], a possible reaction mechanism is proposed (Figure 8) to highlight how the PAM is bound to the sodium alginate structure, and that the sodium alginate is responsible for the performances of SALg-*g*-PAM type as flocculant.

Thus, the FTIR spectrum of polyacrylamide displays principally large bands due to the asymmetric and symmetric NH_2_ stretching vibrations in the regions of 3350–3330 and 3200–3190 cm^−1^ and due to asymmetric and symmetric CH_2_ stretching vibrations in the region of 2930–2900 cm^−1^, respectively. Additionally, the methylene group vibrations were used to evaluate the extension of polymerization of acrylamide as it is presented in Figure 8. The appearance of bands in the regions 1654–1645, 1620–1605, and 1450–1415 cm^−1^ can be attributed to the stretching of the C=O group (amide I), NH_2_ bending (amide II), and C–N stretching vibrations (amide III), respectively. 

The sodium alginate spectrum exhibits mainly bands due to: The Na−O stretching at 815–800 cm^−1^, the stretching frequency of the –OH group at 3400–3000 cm^−1^, the COO– group at 1610–1605 cm^−1^ and 1417–1415 cm^−1^, and the C−O group [25,47].

In the sodium alginate-*g*-acrylamide spectrum, all the absorption bands that were mentioned above are shifted, indicating that the interaction between the functional groups of sodium alginate and polyacrylamide occurs. The bands at 2942–2933, 1608–1605, 1418–1414, and 1034–1031 cm^−1^ presented in the mentioned spectra indicate the stretching vibrations of aliphatic C–H, COO– (asymmetric), COO– (symmetric), and C–O [56]. Moreover, the band located at 3192–3190 cm^−1^ that is due to the OH stretching vibration [25] shows variations in intensity depending on the irradiation dose, which indicates the participation of hydroxyl groups in the chemical reaction, as shown in Figure 8. More than that, some additional bands in the copolymer spectrum are at 1651–1648, 1614–1605, and 1415–1414 cm^−1^ which correspond to the carbonyl amide, N–H, and C–N stretching vibrations [25,47]. All these bands have confirmed the grafting of polyacrylamide on the sodium alginate backbone, as in Figure 8. 

### 3.3. Flocculation Study Results

Grafted polymers characterized as above were used in flocculation studies that were carried out on kaolin suspension at room temperature of 25 °C. The influence of kaolin concentration (0.05, 0.1, and 0.2 wt %), polymer concentration (0.5 to 10 ppm), and rotation speed (60 and 90 rpm) on flocculation efficacy in terms of transmittance against distilled water were studied. 

**Figure 9:**
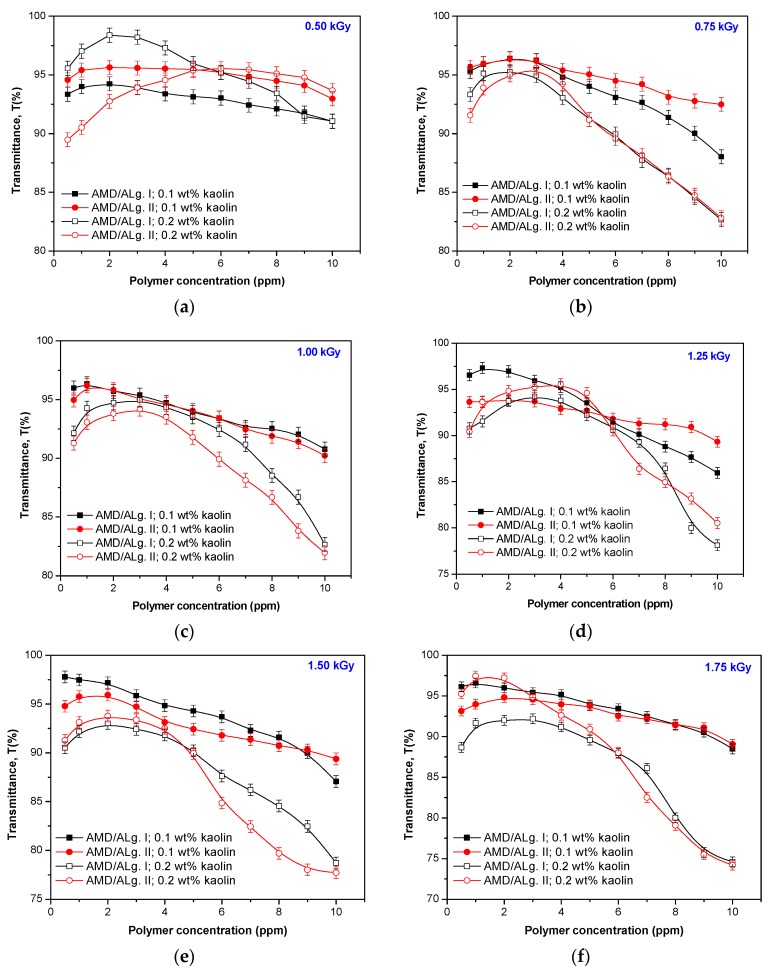

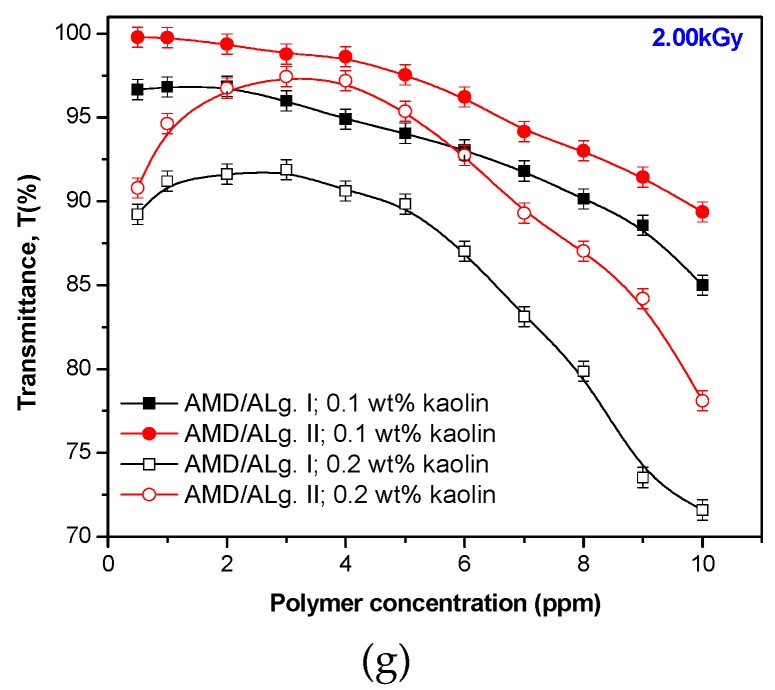
The influence of the polymer type and kaolin concentration on the samples transmittance: AMD/ALg I and II obtained at 0.5 kGy (**a**), 0.75 kGy (**b**), 1 kGy (**c**), 1.25 kGy (**d**), 1.5 kGy (**e**), 1.75 kGy (**f**), and 2 kGy (**g**).

For the first experiment set, in the Jar test glass bakers filled with 500 mL kaolin suspension of 0.1 and 0.2 wt % were added polyelectrolytes of AMD/ALg I and AMD/ALg II types in different concentrations between 0.5 and 10 ppm. Samples were stirred first at 60 rpm for 15 min, then left to rest for another 15 min before sampling for analysis from the top layer clear supernatant. The results are presented in Figure 9.

As seen in Figure 9, the increase of the polymer concentration over 4 ppm leads to the obtainment of transmittances that are increasingly small, irrespective of the irradiation dose at which the polymers were obtained or of the concentration of the kaolin suspension. In the range of polymer concentration from 1 to 4 ppm, transmittances over 90% were obtained for both 0.1 and 0.2 wt % kaolin suspensions.

In the experiments that were made on the kaolin suspension of 0.1 wt %, the influence of the sodium alginate concentration in the polymer sample was as follows:

1. Using polymers of AMD/ALg I type in concentration of 1 ppm, transmittances over 96% were obtained (Figure 9b–g), except in the case of AMD/ALg I/0.5;
2. Using polymers of AMD/ALg II type in concentrations of 0.5 and 1 ppm, higher net transmittances were obtained in the case of AMD/ALg II/0.5 (over 98%, Figure 9a) and AMD/ALg II/2 (100%, Figure 9g);
3. Considering as being of interest the concentration range between 0.5 and 4 ppm, it can be observed that even if the polymers of AMD/ALg II types physicochemical properties are inferior to those of AMD/ALg I types, especially in terms of intrinsic viscosity/molecular weight, the best flocculation results were obtained in the case of their use (AMD/ALg II/2 having intrinsic viscosity of 1.283 dl/g versus AMD/ALg I/1 having intrinsic viscosity of 2.081 dl/g);

In the experiments that were made on the kaolin suspension of 0.2 wt %, the influence of the sodium alginate concentration in the polymer sample was as follows:

1. Irrespective of the polymer type used, the maximum transmittance did not reach 100%. However, there were some cases in which over 97% was obtained: AMD/ALg I/0.5, AMD/ALg II/1.75 and AMD/ALg II/2 for polymer concentrations of 2 ppm (Figure 9a), 1 ppm (Figure 9f), and 3 ppm (Figure 9g);
2. Except in the cases of AMD/ALg I/0.5, AMD/ALg I/0.75, and AMD/ALg I/1, the polymers of AMD/ALg II types were more efficient than those of AMD/ALg I types. Thus, I can be said that for more concentrated kaolin suspensions, high sodium alginate concentrations are needed, and for AMD/ALg II polymer types, higher irradiation doses are necessary;
3. In order to obtain similar results, polymers of AMD/ALg I type have to be used in lower concentrations than polymers of AMD/ALg II type.

**Figure 10:**
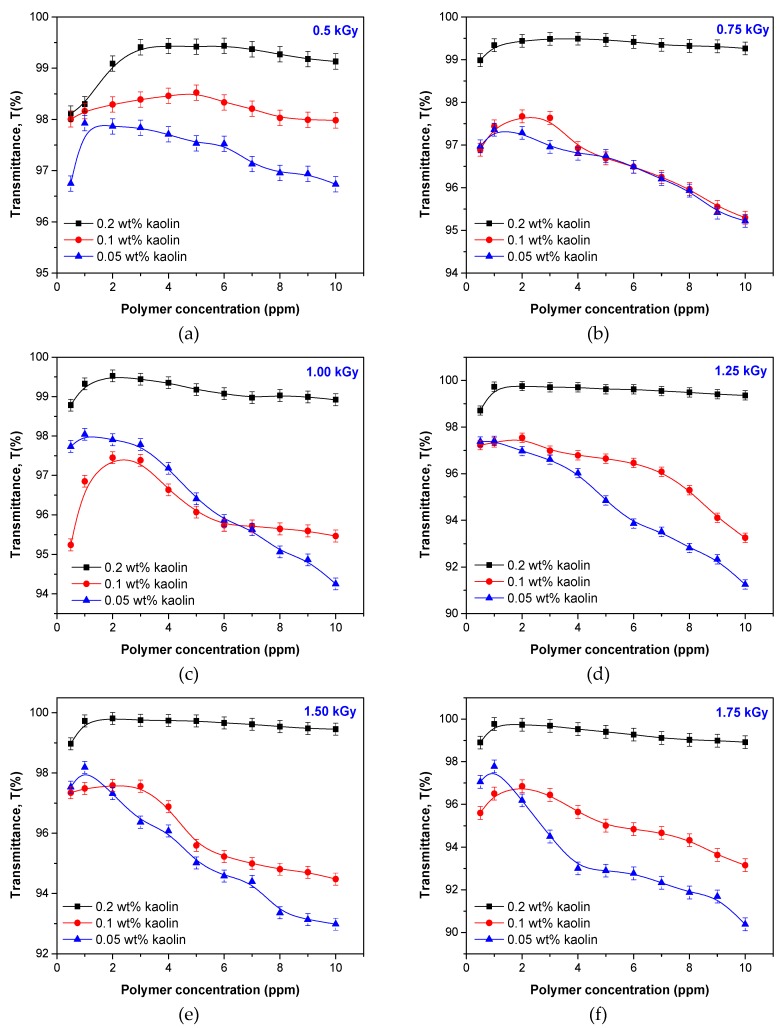

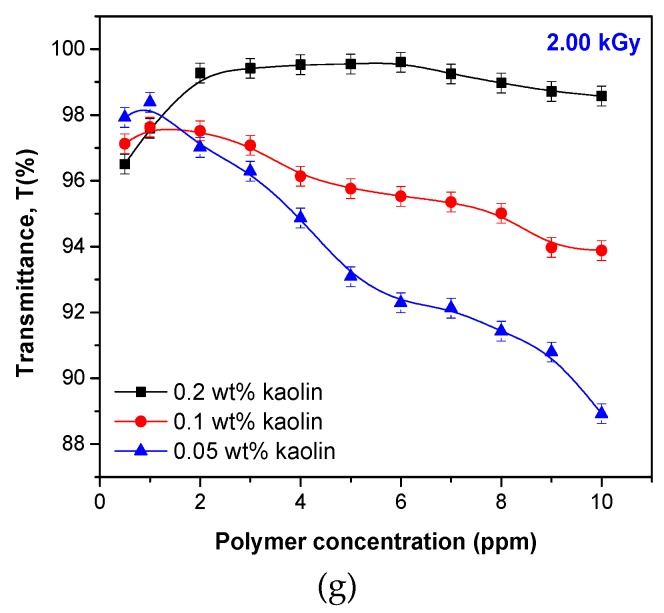
The influence of the kaolin concentration on the samples transmittance: AMD/ALg II obtained at 0.5 kGy (**a**), 0.75 kGy (**b**), 1 kGy (**c**), 1.25 kGy (**d**), 1.5 kGy (**e**), 1.75 kGy (**f**), and 2 kGy (**g**).

For the second experiment set, the polymers of AMD/ALg II type, having lower intrinsic viscosity, grafting degree, and grafting efficiency, were deliberately chosen. The experiments were done almost in the same way, the only differences consisting of the kaolin suspension concentration (0.05, 0.1 and 0.2 wt %) and rotation speed (90 rpm). The results are presented in Figure 10.

As seen in Figure 10, as the kaolin suspension is more concentrated, the polymers having higher sodium alginate content obtained at the irradiation dose between 0.75 and 1.75 kGy are more efficient. For the kaolin suspension of 0.2 wt %, the polymer concentration of 1 ppm is enough to obtain transmittances over 99%. For the kaolin suspensions of 0.05 and 1 wt %, the same tendency as in the first experiments set appeared. Only small polymer concentrations (around 1 ppm for 0.05 wt % and 2 ppm for 1 wt %, except for AMD/ALgII/0.5 and AMD/ALgII/1.5) led to the obtainment of transmittances up to 98%. By increasing the polymer concentration, the flocculation results were depreciated for both the 0.05 and 1 wt % kaolin suspension but keeping the tendency for the worst results as the kaolin concentration decreases.

Many authors developed bridging theories that provide the abilities of polymers having high molecular weights [57,58,59] and grafting ratios [60,61] to destabilize colloidal suspensions. Destabilization by bridging occurs when segments of a polymer chain adsorb one or more particles, thereby linking the particle together. When a polymer molecule comes into contact with a colloidal particle, some of the reactive groups of the polymer adsorb at the particle surface, leaving other portions of the molecule extending in the solution. The polymer will adsorb on the surface in a series of loops and trails. If a second particle with some vacant adsorption sites contacts the extended loops and trails, attachment can occur. A particle–polymer–particle aggregate is formed, in which the polymer serves as a bridge [58,60].

Because our flocculation experiments were carried out in neutral conditions (the pH of kaolin suspension was of 7) and based on experimental facts regarding the aspect of the formed aggregates, we consider that the flocculation mechanisms that occurred in our experiments were based on bridging, first because this mechanism is dominant in neutral and alkaline conditions [58,60], and second, because of the compact aspect of the flocs. 

### 3.4. Metal Ion Removal Study Results

Metal ion removal experiments were done at room temperature in aqueous solutions containing concentrations of 500 mg/L Cu^2+^ and Cr^6+^ ions. High initial concentrations usually provide the driving forces necessary to overcome mass transfer resistance of a metal ion between the aqueous solution and the solid and increase the metal uptake. In addition, a high initial concentration increases the number of collisions between metal ions and sorbent, which enhances the sorption process [23,62,63]. 

**Figure 11:**
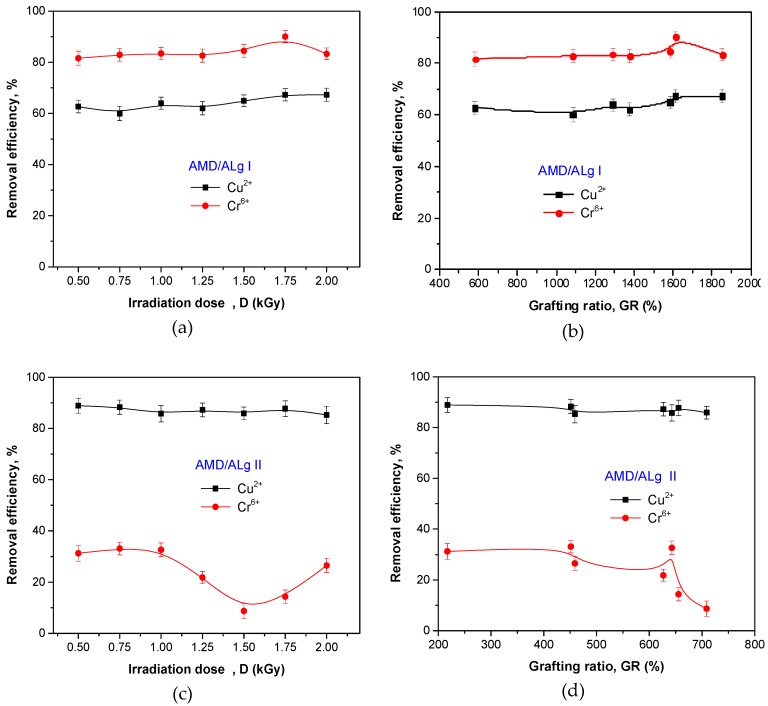
The influence of irradiation dose, grafting ratio, and sodium alginate concentration on Cu^2+^ and Cr^6+^ ion removal efficiency. (**a**) removal efficiency versus irradiation dose for AMD/ALg I samples; (**b**) removal efficiency versus grafting ratio for AMD/ALg I samples; (**c**) removal efficiency versus irradiation dose for AMD/ALg II samples; (**d**) removal efficiency versus grafting ratio for AMD/ALg II samples.

**Figure 12:**
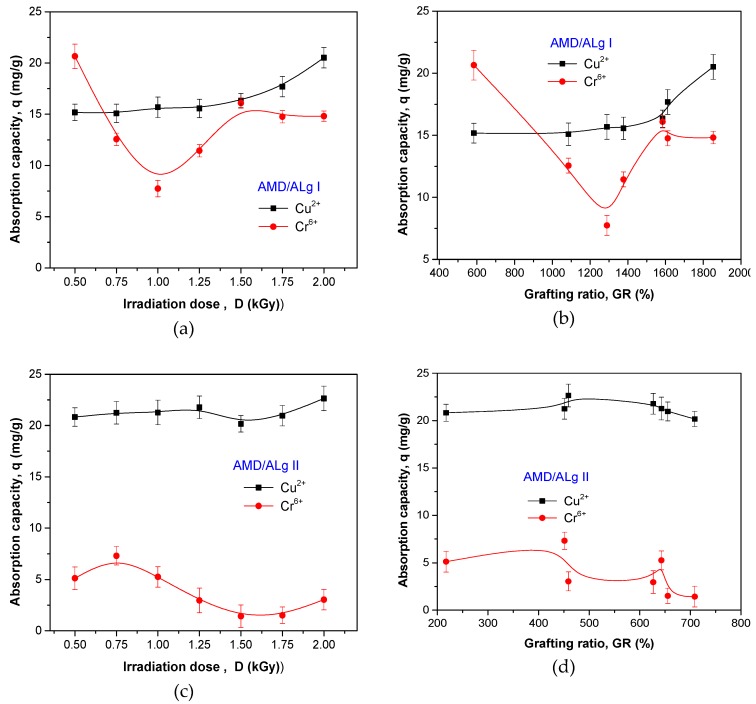
The influence of irradiation dose, grafting ratio, and sodium alginate concentration on equilibrium absorption capacity. (**a**) absorption capacity versus irradiation dose for AMD/ALg I samples; (**b**) absorption capacity versus grafting ratio for AMD/ALg I samples; (**c**) absorption capacity versus irradiation dose for AMD/ALg II samples; (**d**) absorption capacity versus grafting ratio for AMD/ALg II samples.

The flocculant amount of 1g was used in experiments. Graft copolymer samples were immersed for 24 h at room temperature of 25 °C in 20 mL solution of metal ions having a neutral pH of 7. After treatment, samples of metallic solutions were analyzed using a Cary Bio-100 UV–VIS Spectrophotometer in order to determine the residual concentration. The influence of the grafting conditions (irradiation dose) and sodium alginate concentration on removal efficiency and absorption capacity after 24 h was studied. The results are presented in Figure 11 and Figure 12. 

In Figure 11, it can be seen that irrespective of irradiation dose and grafting ratio, polyelectrolyte samples of AMD/ALg I type were over 80% efficient in Cr^6+^ removal and over 60% in Cu^2+^ removal, while samples of AMD/ALg II type were near 90% efficient in Cu^2+^ removal and between 10 and 30% efficient in Cr^6+^ removal. In the case of the AMD/ALg II type, the increase of the irradiation dose and grafting ratio led to the obtainment of polymers that are less efficient in Cr^6+^ ion removal. 

In Figure 12a,b, it can be seen that after 24 h, the absorption capacities of both Cr^6+^ and Cu^2+^ ions calculated for AMD/ALg I polymer type are dependent on the irradiation dose and grafting ratio. The absorption capacity of Cu^2+^ ions was around 15 mg/g irrespective of irradiation dose up to 1.5 kGy and grafting ratio up to 1600%. The increase of the irradiation dose with 0.5 kGy up to 2 kGy is reflected in the increase of the grafting ratio with 400%, which led to an improvement in the absorption capacity of Cu^2+^ ions with almost 30%. 

As seen in Figure 12c,d, the absorption capacity of Cu^2+^ ions presented only small variations around 20 mg/g when the AMD/ALg II polymer type was used, the highest value being obtained for the grafting ratio of 500%. By comparison, the absorption capacity of Cr^6+^ ions is modest, just passing over 5 mg/g for a grafting ratio of 400%.

The results suggest that polyelectrolytes of AMD/ALg I type having high grafting ratios (between 600% and 1900%) are efficient in Cr^6+^ ion removal, while the polyelectrolytes of AMD/ALg II type having small grafting ratios (between 200% and 700 %) are efficient in Cu^2+^ ion removal. The results are comparable to those reported in the literature [23,64].

The experiments for heavy metal ion (Cu^2+^ and Cr^6+^) removal using exclusive sodium alginate were performed by introducing 0.1 grams (experiment *a*) and 0.2 grams (experiment *b*) in 20 mL of 500 mg/L Cu^2+^ and Cr^6+^ ions. The sodium alginate in the chrome solution was totally dissolved; thus, the spectrophotometric measurements could not be done. The sodium alginate in the cooper solution did not dissolve after 24 h and spectrophotometric measurements were performed. The absorption capacities were of 3.62 mg of cooper per gram of sodium alginate in experiment *a* and of 30.5 mg of cooper per gram of sodium alginate in experiment *b*. These results could not be compared with those presented in Figure 11 and Figure 12, where the results were reported for every gram of grafted polymer. Even if the copolymers of AMD/Alg I and AMD/Alg II type contain, in each gram, 0.01 grams and 0.02 grams respectively of sodium alginate, they were more efficient in cooper removal than the alginate alone.

Even if, generally, the initial heavy metal ions concentration is very low in most wastewaters [64], it is necessary to continue the development of new types of polymeric materials applicable for wastewaters coming from very high polluting industries with heavy metals (textile, leather, metallurgical, etc.). Hence, the results obtained in our experiments are important because they have shown high heavy metal ion removal capacities using low flocculant concentration. 

Additionally, in the literature, satisfactory results are presented regarding heavy metal removal using superabsorbent hydrogels, but it is hard for them to be applied to existing technological systems. Most water purification systems use the currently flocculation method with polyelectrolytes, like a studied product. 

## 4. Conclusions

Two types of sodium alginate-*g*-acrylamide polyelectrolytes (AMD/ALg I and AMD/ALg II containing 1% and 2% sodium alginate, respectively) were obtained by electron beam irradiation in the range of 0.5 to 2 kGy. The grafting of acrylamide onto the sodium alginate backbone was evaluated and highlighted by physical, chemical, and structural analysis. The differences of 1500% between the grafting ratios and of 30% between the grafting efficiencies of the grafted products wave showed that the irradiation dose over 1.5 kGy became critical when the sodium alginate concentration was doubled. The grafted products were used in flocculation and heavy metal ion removal studies. The polyelectrolyte type, concentration, rotation speed, and contact time were found to be of a notable importance on flocculation efficacy. Even the grafting ratio of AMD/ALg II type was only up to 500%, the polyelectrolytes of this type were more efficient than the AMD/ALg I type used in the same concentration, as the kaolin concentration increased. However, by decreasing the polyelectrolyte concentration at 1 ppm and increasing the rotation speed to 90 rpm, the AMD/ALg I type became more efficient and transmittances against distilled water near 100% were obtained. The heavy metal ion removal studies show that the polyelectrolytes of AMD/ALg I type are more efficient in Cr^6+^ ion removal, while the polyelectrolytes of AMD/ALg II type in Cu^2+^ ion removal. Both the removal efficiency and absorption capacity results suggest that the irradiation dose is critical for obtaining efficient polyelectrolytes for Cr^6+^ ion removal.

## Figures and Tables

**Table 1:** The materials used for polyelectrolytes preparation.

Materials	Chemical Characteristics	Chemical Structure/Molecular Formula
Acrylamide, AMD	molecular weight: 72.06 g/mol; density: 1.051 g/cm^3^; solubility in water: 2.04 kg·L^−1^ at 25 °C;	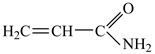
Potassium persulfate, PP (used as reaction initiator)	molecular weight: 270.322 g/mol; density: 2.477 g/cm^3^; solubility in water: 1.75 g/100 mL at 0 °C;	K_2_S_2_O_8_
Sodium alginate, ALg	molecular weight: 216.121 g/mol; density: 1.601 g/cm^3^ solubility in water: no more than 2% on the dried basis	(C_6_H_7_NaO_6_)_n_ or C_6_H_9_NaO_7_

**Table 2:** The polyelectrolytes synthesis details.

Samples Codes	Amount of Chemicals			Irradiation Dose (kGy)
	AMD (mol/L)	Alg (%)	PP (mol/L)	
AMD/ALg I/0.5	2.63	1	9.25 × 10^−4^	0.5
AMD/ALg I/0.75				0.75
AMD/ALg I/1				1
AMD/ALg I/1.25				1.25
AMD/ALg I/1.5				1.5
AMD/ALg I/1.75				1.75
AMD/ALg I/2				2
AMD/ALg II/0.5	2.63	2	9.25 × 10^−4^	0.5
AMD/ALg II/0.75				0.75
AMD/ALg II/1				1
AMD/ALg II/1.25				1.25
AMD/ALg II/1.5				1.5
AMD/ALg II/1.75				1.75
AMD/ALg II/2				2

**Table 3:** Physical and chemical characteristics of sodium alginate-*g*-acrylamide polymers. AMD/ALg I (I) and AMD/ALg II (II).

Dose (kGy)	*C*_c_ (%)		*M*_rez._ (%)		η_intr._ (dl/g)	
	I	II	I	II	I	II
**0.5**	83.21	83.04	0.0604	0.0675	1.516	0.822
**0.75**	90.76	90.41	0.0355	0.0369	1.880	1.172
**1**	92.54	92.99	0.0277	0.0269	2.081	1.790
**1.25**	93.64	93.94	0.0241	0.0227	1.625	1.632
**1.5**	95.39	94.69	0.0171	0.0199	1.491	1.532
**1.75**	96.97	96.51	0.0114	0.0135	1.469	1.301
**2**	96.58	96.72	0.0128	0.0128	1.403	1.283
